# Increased nasal matrix metalloproteinase-1 and -9 expression in smokers with chronic rhinosinusitis and asthma

**DOI:** 10.1038/s41598-019-51813-6

**Published:** 2019-10-25

**Authors:** Chien-Chia Huang, Chun-Hua Wang, Pei-Wen Wu, Jung-Ru He, Chi-Che Huang, Po-Hung Chang, Chia-Hsiang Fu, Ta-Jen Lee

**Affiliations:** 1 Division of Rhinology, Department of Otolaryngology, Chang Gung Memorial Hospital and Chang Gung University, Taoyuan, Taiwan; 2 Graduate Institute of Clinical Medical Sciences, College of Medicine, Chang Gung University, Taoyuan, Taiwan; 3 Department of Thoracic Medicine, Chang Gung Memorial Hospital and Medicine of College, Chang Gung University, Taoyuan, Taiwan; 4 Department of Otolaryngology–Head and Neck Surgery, Chang Gung Memorial Hospital and Chang Gung University, Keelung, Taiwan; 5 Department of Otolaryngology, Xiamen Chang Gung Hospital, Xiamen, China

**Keywords:** Asthma, Prognostic markers

## Abstract

A potential mechanism underlying cigarette smoke-induced airway disease is insufficient tissue repair via altered production of matrix metalloproteinases (MMPs). Osteitis is a signature feature of recalcitrant chronic rhinosinusitis (CRS) and often results in revision surgery. The present study aimed to investigate MMP expression in the nasal tissues of asthmatic patients with CRS and any association with cigarette smoking and osteitis. Thirteen smokers with CRS and asthma, 16 non-smokers with CRS and asthma, and seven non-smoker asthmatic patients without CRS were prospectively recruited. The expression of MMPs and associated immunological factors in surgically-obtained nasal tissues was evaluated via real-time PCR and western blotting. Maximal bone thickness of the anterior ethmoid (AE) partition was measured in axial sinus computed tomography (CT) sections. MMP-1 and MMP-9 expression was increased in the nasal tissues of smokers with asthma and CRS via real-time PCR and western blot. Maximal AE partition bone thickness was greater in smokers with CRS and asthma than in non-smokers with CRS and asthma. MMP-1 and MMP-9 levels were correlated with maximal AE bone thickness. Cigarette smoking was associated with the up-regulation of MMP-1 and MMP-9 in the nasal tissues of patients with airway inflammatory diseases, and with AE osteitis, and with therapeutic resistence.

## Introduction

Chronic rhinosinusitis (CRS) and asthma are common inflammatory airway diseases and frequently comorbid, as per the “unified airway” concept^1^. Type 2 inflammatory cytokines, such as IL-5 and IL-13, are considered key drivers of airway inflammation in patients with CRS and asthma^2,3^. However, the IL-17A mediated immune response has been linked to neutrophil recruitment, airway remodeling, and resistance to corticosteroid-based therapy in airway disease cases^4–6^. A recent study by our group revealed that cigarette smoking was related to IL-17A activation in the nasal tissues of asthmatic patients with CRS and attenuated improvements in asthmatic patients after nasal surgery^7,8^. Cigarette smoke contains over a 1,000 chemicals, and chronic obstructive pulmonary disease (COPD)-like airway injury and remodeling occur in chronic cigarette smoke exposure rodents models^9,10^. A potential mechanism for cigarette smoke-induced airway disease is insufficient tissue repair via altered production of matrix metalloproteinases (MMPs)^11^.

MMPs comprise a family of Ca^2+^-activated, zinc-dependent endopeptidases, which may be produced by airway epithelial cells, fibroblasts, and inflammatory cells^11,12^. MMPs play an essential role in the degradation of basement membranes and extracellular matrix proteins including collagens IV, V, VII, X, and XIV; gelatin; and elastin. MMPs contribute to the tissue edema and remodeling seen frequently in inflammatory diseases of the airway including asthma, COPD, and CRS^11,13,14^.

Tissue remodeling in the lower airway has been extensively studied in asthmatics and in patients with COPD. For example, MMP-9, also known as gelatinase B or 92 kDa gelatinase, is a critical elastolytic enzyme produced by alveolar macrophages in COPD patients. It is also secreted by neutrophils, epithelial cells, mast cells, and fibroblasts^15^. Higher levels of MMP-9 are associated with increased asthma severity and decreased lung functioning. MMP-12 is also an elastolytic proteinase found in the alveolar macrophages of cigarette smokers^16^ and is relevant to cigarette smoke-induced emphysema^17^.

Recent studies have indicated that sinonasal tissue remodeling occurs secondary to CRS^18^. Elevated MMP-1,-2,-7,-8, and -9 have also been noted in the sinonasal tissues of CRS patients^14,19,20^. In particular, MMP-9 is significantly lower in patients with good mucosal healing after sinus surgery compared to those with poor healing^21^. MMP-9 plays an important role in the pathophysiology of osteitis in CRS^22^. Osteitis, a form of neo-osteogenesis due to inflammation rather than infection, is a common factor in recalcitrant CRS and is associated with revision sinus surgeries and CRS severity, indicated by elevated Lund-Mackay computed tomography (CT) scores^23–25^. IL-17A, a signature CRS cytokine^26,27^, promotes MMP-9 expression by activating the NF-κB signaling pathway in the nasal tissues of patients with CRS and nasal polyps^28^. As a result, the relationship between cigarette smoking, IL-17A, and MMPs is worthy of investigation.

Given the above background, we hypothesized that IL-17A-mediated immune responses and MMPs expression might be associated with cigarette smoke-related airway inflammation, osteitis formation, and resistance to current therapeutic regimens. The present study aimed to investigate the expression of MMPs in the nasal tissues of asthmatic patients with CRS and its association with cigarette smoking and osteitis.

## Methods

### Patients

The Institutional Review Board of the Chang Gung Memorial Hospital approved of all study procedures undertaken here (IRB Numbers: 101-5069B and 103-7085B). Asthmatic patients with CRS were prospectively recruited between August 2013 and June 2016. Asthma diagnoses fulfilled Global Initiative for Asthma (GINA) asthma diagnosis guidelines^29^ and CRS was defined using the criteria established in the European CRS position paper^30^ and was based on subjective symptoms and objective findings from nasal endoscopy and sinus CT. Participant inclusion criteria included a diagnosis of asthma (1) with regular follow-up for at least 1 year; (2) failed medical treatment for nasal symptoms; and (3) plans to undergo nasal surgery. Exclusion criteria included previous nasal surgeries or major medical comorbidities such as diabetes, nephrotic diseases, autoimmune disorders, immunodeficiencies, malignancies, and other chronic illnesses. Seven non-smoker asthmatic patients without CRS (confirmed by sinus CT) were enrolled in the control group during septomeatoplasty and/or turbinoplasty. Participants provided informed consent prior to study enrollment. All research was performed in accordance with the relevant guidelines and regulations and all Institutional Review Board requirements.

Patient clinical characteristics were determined at enrollment and at a 6 month follow-up appointment. Patients were asked if they regularly used cigarettes or other forms of tobacco pre-operatively. Patients were considered to be smokers if they reported regular or on-going smoking in the last 12 months and non-smokers were considered to be those who never regularly used cigarettes^31^.

**Figure 1:**
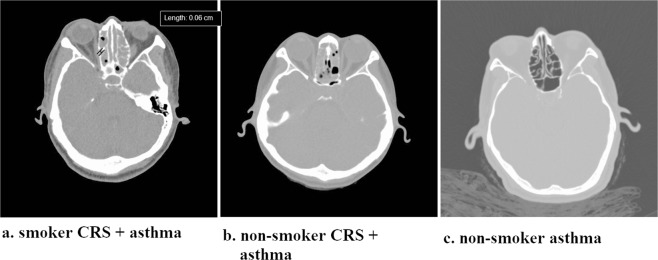
Non-contrast sinus computed tomography (CT). The maximal thickness of the bony partition of the anterior ethmoid sinuses (indicated by caliper) was measured on an axial sinus CT section. caliper

A preoperative nasal endoscopy examination and sinus CT, which were scored using the Lund-Kennedy endoscopy score and Lund-Mackay CT score, were completed in all patients. The maximal thickness of the bony partition of the anterior ethmoid sinuses (AE) was measured on an axial sinus CT section for each patient (Fig. 1). Pulmonary function tests, including forced vital capacity (FVC), forced expiratory volume in 1 second (FEV_1_), and FEV_1_/FVC ratio, were measured before and 6 months after nasal surgery. The severity of sinonasal symptoms and the level of asthma control were evaluated by the Sino-Nasal Outcome Test-22 (SNOT-22)^32^ and Asthma Control Test (ACT) questionnaires^33^, respectively.

### Collection and processing of specimens

Nasal mucosal specimens were obtained during nasal surgery and rinsed in phosphate-buffered saline (pH 7.6), stored in liquid nitrogen at −70 °C, and then processed for immunoblotting and real-time polymerase chain reaction (PCR).

### Real-time PCR

Frozen nasal tissues from all 36 participants were homogenized (Retsch, Haan, Germany). Total RNA was extracted from nasal specimens using the RNeasy Mini Kit (Qiagen, Strasse, Germany) and quantified by NanoDrop (Thermo Scientific, Barrington, Ill). Reverse transcription was performed with random hexamer primers using the High-Capacity cDNA Reverse Transcription Kit (Applied Biosystems, Foster City, CA, USA). Real-time PCR was done using the TaqMan assay with primers specific to each target gene (Table 1) and glyceraldehyde-3-phosphate dehydrogenase (GAPDH) was used as the internal housekeeping control gene. The Applied Biosystems 7500 Fast Real-Time PCR System (Applied Biosystems) was used. The amplification conditions were an initial incubation at 95 °C for 10 min; 45 cycles of 95 °C for 10 s, 60 °C for 20 s, and 72 °C for 10 s; and final cooling to 40 °C. Each sample was run in triplicate. All triplicate mean threshold cycle (Ct) values were normalized to GAPDH to determine the ΔCt values and relative mRNA levels for each target gene were calculated using the ΔΔCt method.

**Table 1:** Primer sequences specific to target genes.

	Forward primers	Reverse primers
IL-5	AGACCTTGGCACTGCTTTCT	CAGTACCCCCTTGCACAGTT
IL-13	AGACCTTGGCACTGCTTTCT	CAGTACCCCCTTGCACAGTT
IL-17A	TTGGTGTCACTGCTACTGCT	TTGGGCATCCTGGATTTCGT
AhR	TTGTGCCGAGTCCCATATCC	TGGCAGGAAAAGGGTTGGTT
MMP-1	ATG CTT TTC AAC CAG GCC CA	AGT CCA AGA GAA TGG CCG AG
MMP-9	CGC AGA CAT CGT CAT CCA GT	AAA CCG AGT TGG AAC CAC GA
MMP-12	GAC CTG GAT CTG GCA TTG GAG	AGC AGA GAG GCG AAA TGT GTT
GADPH	TTCCAGGAGCGAGATCCCT	CACCCATGACGAACATGGG

IL, interleukin; AhR, aryl hydrocarbon receptor; MMP, matrix metalloproteinase; GAPDH, glyceraldehyde-3-phosphate dehydrogenase.

### Western blot analysis of MMPs

Nasal tissues from 21 participants with adequate specimens, including seven smokers with asthma and CRS, nine non-smokers with asthma and CRS, and five asthmatic non-smokers without CRS were processed for western blot analyses of MMPs. Total protein was prepared using a 1% IGEPAL lysis buffer (Sigma-Aldrich. St. Louis, MO, USA). Cellular proteins (10 μg) were fractionated by SDS-PAGE and electroblotted onto polyvinylidene difluoride membranes. Primary antibodies including MMP-1 (1:200; Millipore, Billerica, MA, USA), MMP-9 (1:1000; CST, MA, USA), MMP-12 (1:100; Santa Cruz Biotechnology, Dallas, TX, USA), and β-actin (1:20,000; Millipore, Billerica, MA, USA) were used to assess differences in protein levels by enhanced chemiluminescence. Protein bands were visualized using a gel documentation system (Alpha Innotech, San Leandro, CA. USA). Relevant band intensities were quantified via densitometric analyses and normalized to β-actin.

### Statistical analyses

Data are presented as mean ± standard deviation (SD) and statistically analyzed using GraphPad Prism 5 (GraphPad Prism Software, Inc, San Diego, Calif). Categorical variables were compared using *х*^2^ or Fisher’s exact tests, as appropriate. Continuous variables were analyzed using the Mann-Whitney U test or Kruskal-Wallis H test when comparing between two or three groups. Correlations were determined using the Spearman’s correlation coefficient (r). Statistical significance was set at p < 0.05.

## Results

### Participant characteristics

Participants included 13 smokers, 16 non-smokers, and 7 non-smokers with asthma and without CRS. Participant characteristics are summarized in Table 2. There were no differences in participant age, severity of sinusitis, presence of nasal polyps, and lung function between smokers and non-smokers with asthma and CRS. Sex differed between the groups, with significantly more males in the smoker group (P < 0.01).

**Table 2:** Clinical characteristics of the study participants.

	Asthma and CRS		Asthma	P value^*^
	Smoker (n = 13)	Non-smoker (n = 16)	Non-smoker (n = 7)	
Age, years	50.9 ± 13.1	49.4 ± 12.9	61.4 ± 9.6	ns
Female: Male, n	1: 12	10: 6	2: 5	<0.01^†^
Atopy(n)	7	10	3	ns
Nasal polyp, n (%)	2 (15.4)	4 (25.0)	0 (0)	ns^†^
CT score	9.2 ± 4.0	9.9 ± 7.1	0.0 ± 0.0	ns^†^
Endoscopy score	4.6 ± 3.1	3.8 ± 3.5	0.9 ± 1.6	ns^†^
Serum IgE (IU/ml)	476.5 ± 871.5	598.3 ± 1253	405.9 ± 764.2	ns
WBC (1000/uL)	7.2 ± 1.6	9.1 ± 4.5	7.2 ± 3.6	ns
Absolute eosinophil (/uL)	186.3 ± 139.6	290.5 ± 303.5	5.0 ± 1.6	ns
SNOT-22	61.5 ± 31.8	55.5 ± 28.9	71.2 ± 30.6	ns
ACT	20.0 ± 4.7	18.6 ± 6.8	19.4 ± 7.0	ns
FVC (% predicted)	76.2 ± 17.8	76.4 ± 15.3	79.1 ± 24.1	ns
FEV_1_ (% predicted)	68.9 ± 15.5	67.3 ± 19.2	61.6 ± 16.5	ns
FEV_1_/FVC (%)	75.5 ± 18.6	79.6 ± 13.6	75.7 ± 14.2	ns

Data are represented as mean ± SD. CRS, chronic rhinosinusitis; CT, computed tomography; IgE, immunoglobulin E; WBC, white blood cell; SNOT-22, sino-nasal outcome test-22; ACT, asthma control test; FVC, forced vital capacity; FEV_1_, forced expiratory volume in 1 s; L, liter; ns, not significant

^*^Categorical variables were compared using Fisher’s exact test and continuous variables were analyzed using the Mann-Whitney U test or Kruskal-Wallis H test when comparing between two or three groups.

^†^Analyses between smoker and non-smoker with CRS and asthma.

### MMP expression in nasal tissues

**Figure 2:**
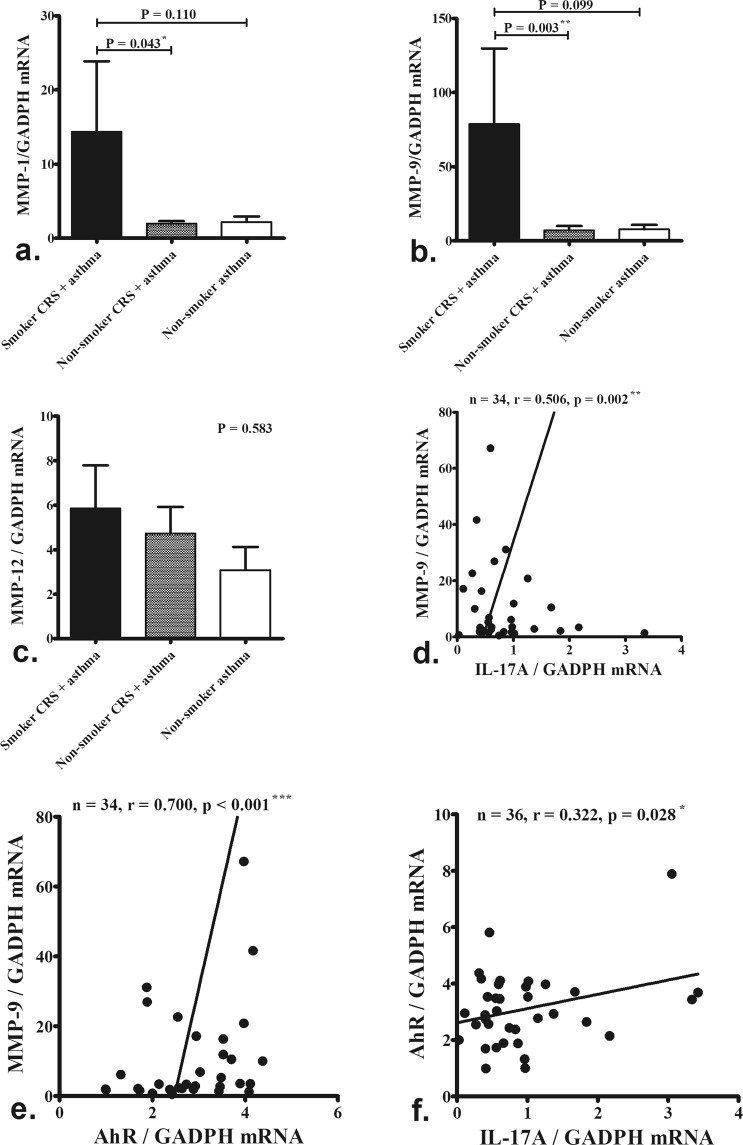
The levels of MMP-1, MMP-9, MMP-12, IL-17A and AhR mRNA expression in nasal tissues. The mRNA expression of MMP-1 (**a**) and MMP-9 (**b**) was significantly up-regulated in the nasal tissues of smokers with CRS and asthma (n = 13) compared to those of non-smokers with asthma and CRS (n = 16). MMP-12 expression did not differ significantly between groups (**c**). MMP-9 mRNA levels were correlated with IL-17A (**d**) and aryl hydrocarbon receptor (AhR) levels (**e**) in nasal tissues. IL-17A mRNA levels were correlated with AhR levels (**f**). The significance is indicated.

The mRNA expression of MMP-1 (Fig. 2a, P = 0.043) and MMP-9 (Fig. 2b, P = 0.003) was significantly up-regulated in the nasal tissues of smokers with CRS and asthma compared to those of non-smokers with asthma and CRS. However, MMP-12 expression did not differ significantly between groups (Fig. 2c, P = 0.583). MMP-9 mRNA levels were correlated with IL-17A (Fig. 2d, P = 0.002) and aryl hydrocarbon receptor (AhR) mRNA levels (Fig. 2e, P <0.001) in nasal tissues. IL-17A mRNA levels were correlated with AhR levels (Fig. 2f, P = 0.028).

**Figure 3:**
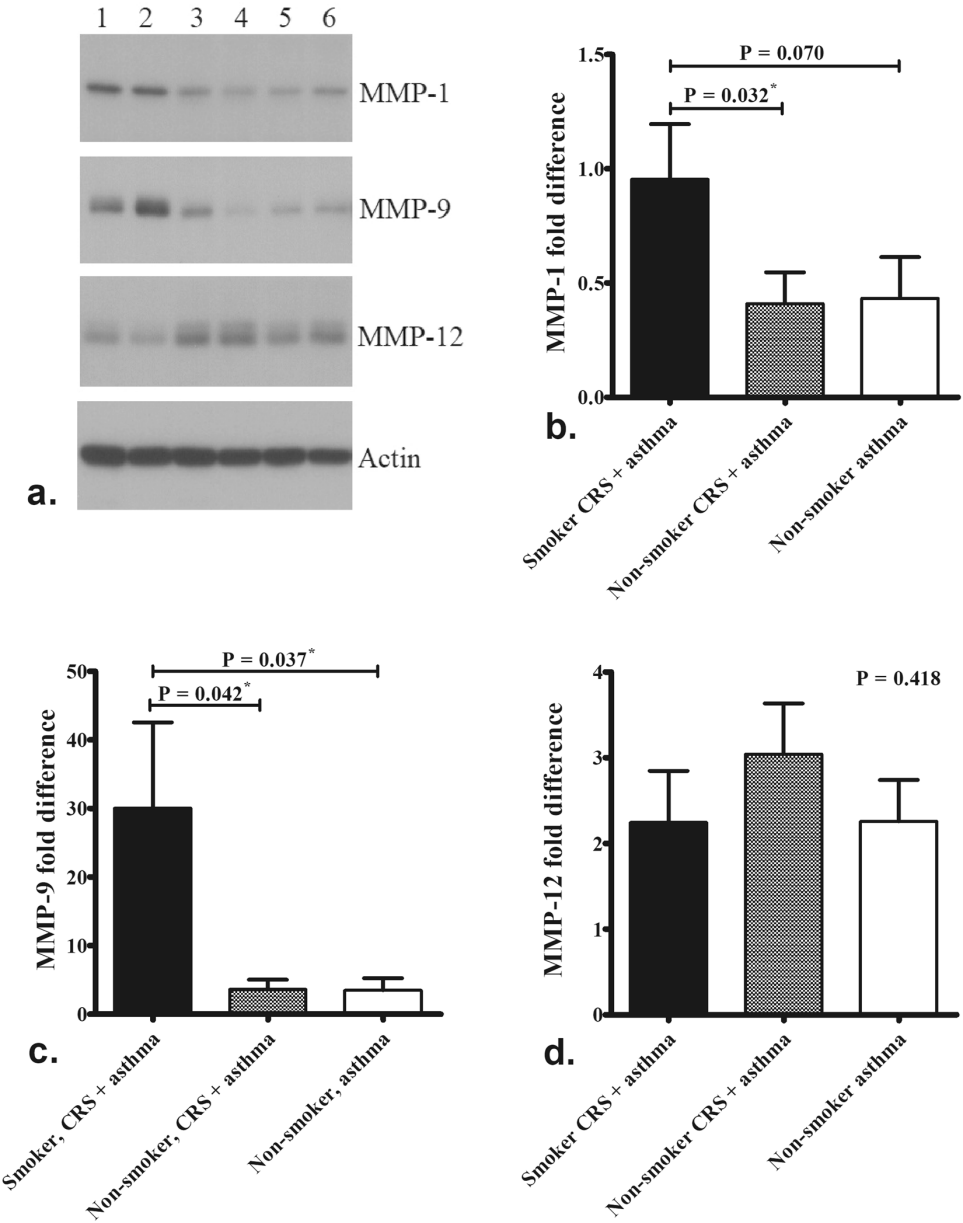
Protein quantification by western blotting. Smokers with CRS and asthma (samples 1 & 2) had higher levels of MMP-1 (**a,b**) and MMP-9 (**a**,**c**) expression than those of non-smokers with CRS and asthma (samples 3 & 4) as well as non-smokers asthma without CRS (sample 5 & 6). There was no difference in MMP-12 expression between the groups (**d**). The grouping of blots cropped from different gels of the same patients. The significance is indicated.

Protein analyzed by western blotting confirmed the results of mRNA and revealed that smokers with CRS and asthma had higher levels of MMP-1 (Fig. 3a,b, P = 0.032) and MMP-9 (Fig. 3a,c, P = 0.042) expression than those of non-smokers with CRS and asthma. There was no difference in MMP-12 expression between the groups (Fig. 3d, P = 0.418).

**Figure 4:**
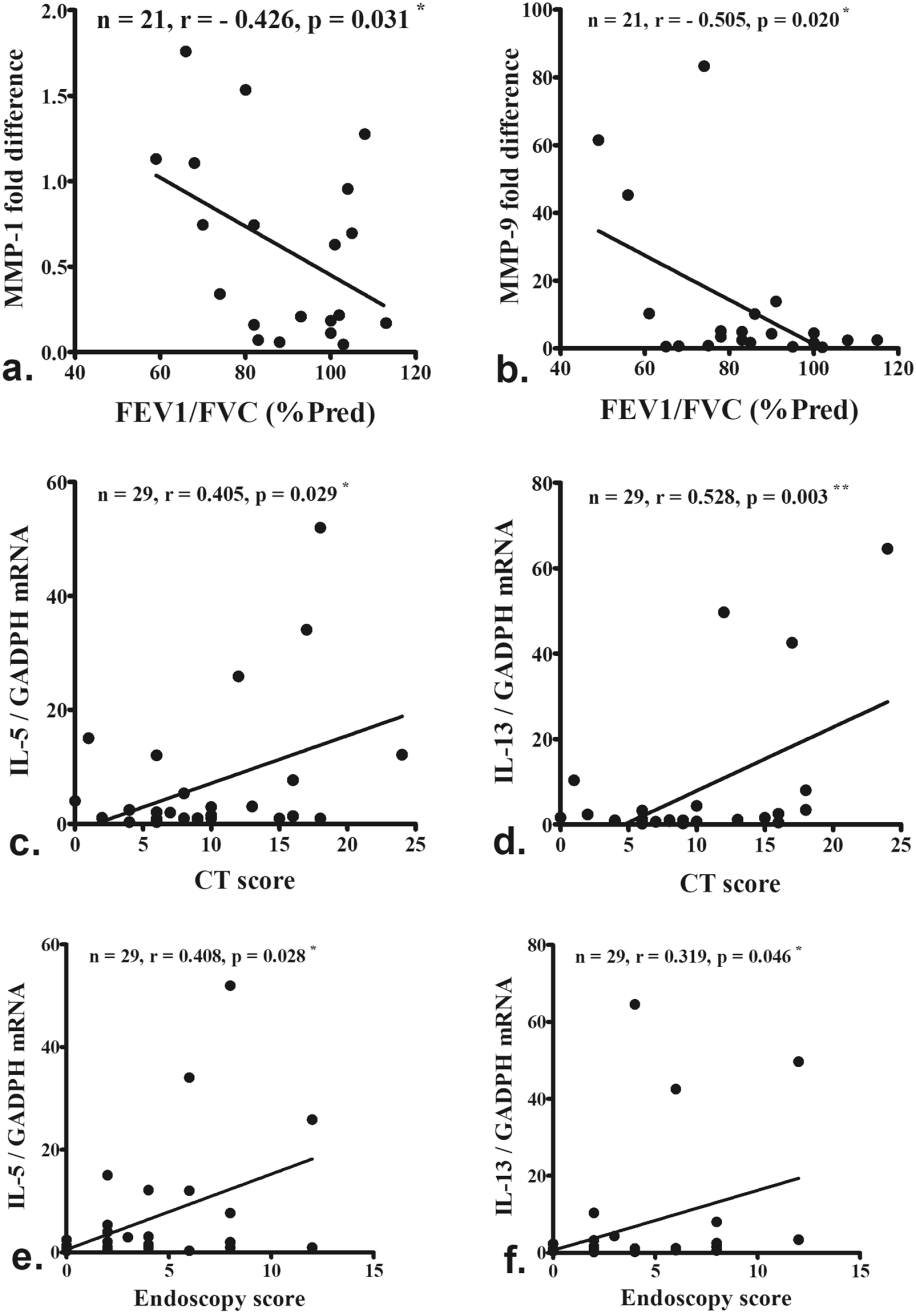
MMP-1 (**a**) and MMP-9 (**b**) protein levels were significantly and inversely correlated with patients’ FEV1/FVC (% predicted). IL-5 and IL-13 mRNA levels were correlated with CT (**c,d**) and endoscopy scores (**e,f**), respectively. The number and significance are indicated.

MMP-1 (Fig. 4a, P = 0.031) and MMP-9 (Fig. 4b, P = 0.020) protein levels were significantly and inversely correlated with patients’ FEV1/FVC (% predicted). IL-5 and IL-13 mRNA levels were correlated with CT (Fig. 4c,d, P = 0.029 and 0.003) and endoscopy scores (Fig. 4e,f, P = 0.028 and 0.046), respectively.

### Maximal AE partition bone thickness on CT

**Figure 5:**
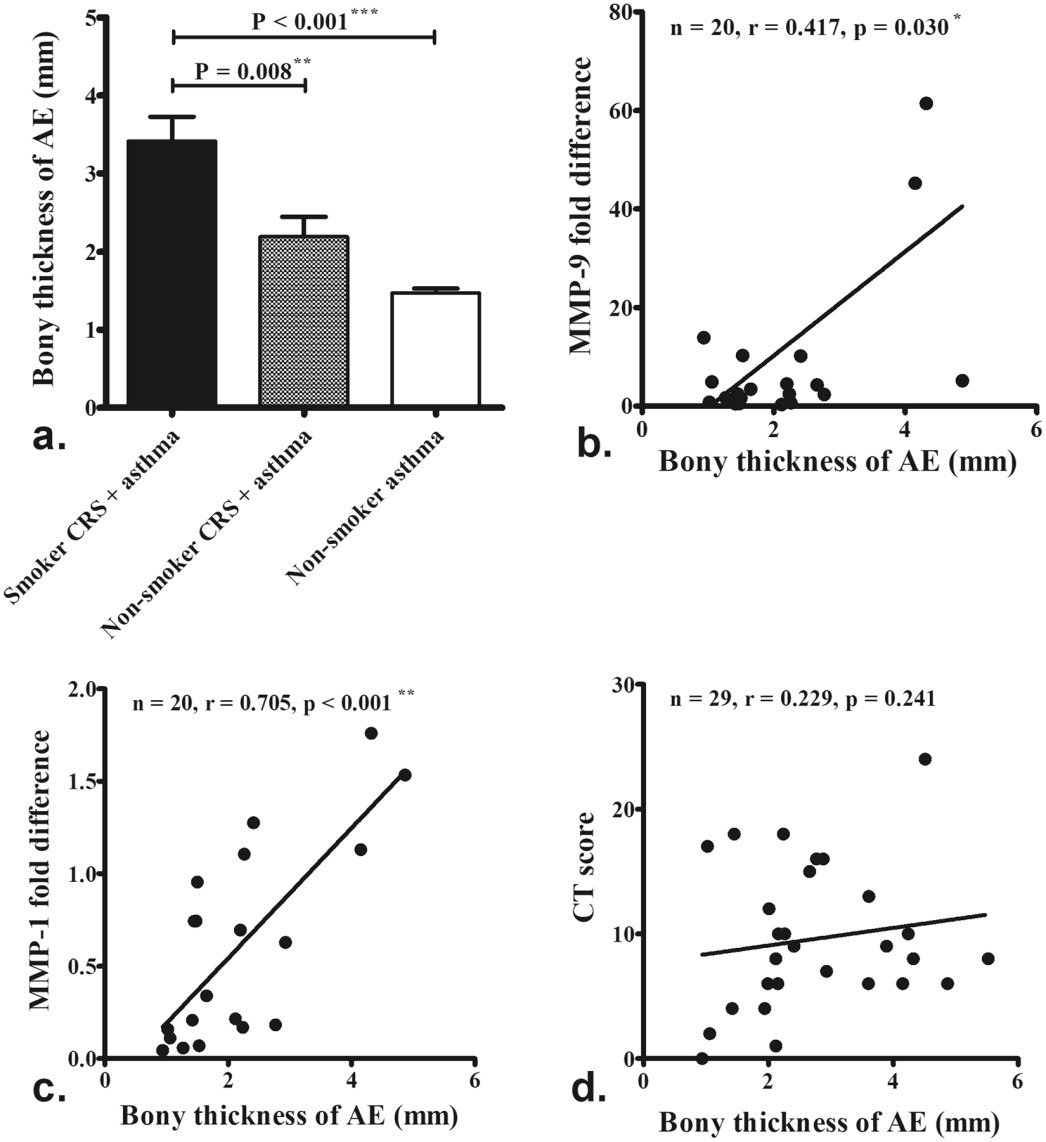
Maximal anterior ethmoid (AE) partition bone thickness measured on axial section sinus CT images was greater in smokers with CRS and asthma than in non-smokers with CRS and asthma, and asthmatic non-smokers without CRS **(a**). MMP-1 (**b**) and MMP-9 (**c**) protein levels were correlated with maximal AE partition bone thickness on CT scans. However, CT scores were not correlated with maximal AE bone thickness (**d**). The number and significance are indicated.

Maximal AE partition bone thickness measured on axial section sinus CT images was greater in smokers with CRS and asthma than in non-smokers with CRS and asthma, and asthmatic non-smokers without CRS (Fig. 5a, P = 0.008 and < 0.001). MMP-1 (Fig. 5b, P = 0.030) and MMP-9 (Fig. 5c, P < 0.001) protein levels were correlated with maximal AE partition bone thickness on CT scans. However, CT scores were not correlated with maximal AE bone thickness (Fig. 5d, P = 0.241).

## Discussion

The present is the first study to evaluate the link between cigarette smoke and AE neo-osteogenesis in patients with asthma and CRS in terms of IL-17A and MMP expression. Our results revealed the expression of MMP-1, MMP-9 in the nasal tissues, and maximal AE bone thickness on CT scans were greater in smokers with asthma and CRS than in asthmatic non-smokers with CRS and asthmatic non-smokers without CRS. Tissue MMP-9 mRNA levels were correlated with IL-17A and the transcription factor AhR. In addition, MMP-1 and MMP-9 protein levels were correlated with maximal AE bone thickness on CT scans.

Osteitis was initially defined as bony thickening of the sinus walls in CRS^34^. The histopathology of osteitis in primary CRS is thought to involve neo-osteogenesis and bone remodeling, which occurs secondary to tissue inflammation^25^. Recent studies have found that osteitis is a characteristic of recalcitrant CRS and is associated with increased disease severity and the need for multiple surgeries^23,24^. Previous studies have also revealed that exposure to smoking diminishes long-term outcomes and increases the risk for revision surgery following endoscopic sinus surgery^31,35^. In the present study, we found a link (involving IL-17A, MMP-1, and MMP-9 expression) between cigarette smoke and neo-osteogenesis in AE with asthma and CRS. Taken together, these results reveal that cigarette smoking may have association with an IL-17A-mediated inflammatory response and increased MMP-1 and MMP-9 expression in the nasal mucosa of patients with asthma and CRS. This may then results in tissue remodeling, which involves neo-osteogenesis or osteitis of the sinus, and thus poorer outcomes following sinus surgery.

Cigarette smoke contains many AhR-activating ligands such as polycyclic aromatic hydrocarbons (PAHs)^36,37^. Exposure to diesel exhaust particles, traffic-related air pollution, and PAH induces IL-17A production and can lead to severe asthma^38^. Th17 polarization is initiated in an AhR-dependent manner through the stimulation of atmospheric particulate matter (PM) in the murine lung. PAHs in the PM are likely sources of Th17-promoting activity^39^. Recent work also demonstrated that IL-17A promotes the expression of MMP-9 by activating the NF-κB signal pathway, revealing a crucial role for IL-17A in the pathogenesis of CRS and associated tissue remodeling^28^. MMP-9 also plays a critical role in the pathophysiology of osteitis in CRS and MMP-9 overexpression, which is steroid-independent, suggesting its relevance to therapeutic resistance^22^. Taken together, MMPs from IL-17A-mediated inflammation via the AhR activation induced by cigarette smoke exposure may thus contribute to neo-osteogenesis and recalcitrant disease in patients with CRS and asthma.

In the current study, MMP-1 and MMP-9 levels were correlated with FEV1/FVC (% predicted), while IL-5 and IL-13 mRNA levels were associated with the CT scores and endoscopy scores, respectively. These results implicate MMPs, which might be induced by cigarette smoke, in lower airway obstruction and sinus osteitis, which were not evaluated via CT and endoscopy scores here. Type 2 inflammatory cytokines such as IL-5 and IL-13 are key mediators of airway inflammation in patients with CRS and asthma^2,3^ and are thus correlated with these objective severity measurements of CT and endoscopy. Furthermore, MMPs such as MMP-1 and -9 may be predictors of lower airway obstruction and resistance to contemporary steroid-based medical treatment in patients with CRS and asthma. Therefore, defining the role of MMPs and their association with immune pathways is imperative in designing, implementing, and tailoring therapeutics to successfully treat these patients. Future novel therapeutics that target MMP-related inflammatory responses may thus be beneficial to those with asthma or CRS that is refractory to current standard treatment protocols. For example, antibiotics such as doxycycline are established MMP-9 inhibitors may be beneficial to this group of patients.

While it offers potentially significant insights into the improved treatment of asthmatic patients with CRS, the present study has several limitations that warrant some consideration. First, this study would have benefited from the inclusion of patients without airway diseases and CRS in a normal control group. However, inclusion of such a group was not permitted by our institutional ethics committee due to the requirement for an invasive biopsy of the nasal mucosa. Second, a larger-scale study is necessary for further subgroup analyses, such as that of CRS patients with and without polyps or patient stratification by the severity of osteitis. Although scoring system of osteitis such as global osteitis scoring scale have been proposed^24^, we suggested it might be suitable for recurrent rather than our primary CRS cases. It not common for bony thickening >3 mm or >5 mm in primary CRS cases. We measured the maximal AE partition bone thickness on CT images to evaluate the severity of neo-osteogenesis instead and AE was selected because it is possible the most exposed sinus to the inhale cigarette smoke. Third, cigarette smoking data was collected by patient self-report, which is not always valid. Quantification of serum nicotine and cotinine levels and analysis of their relationships with cytokine expression should also be further considered to clarify the immune response associated with cigarette smoke exposure of the airway.

Cigarette smoking was associated with the up-regulation of MMP-1 and MMP-9 in the nasal tissues of patients with airway inflammatory diseases and with neo-osteogenesis of AE in the present study.

## Supplementary information

supplementary data

## Data Availability

All data described in this study are presented in the manuscript. All datasets are available from the corresponding author on reasonable request.
