# Supplementary material for: Increased nasal matrix metalloproteinase-1 and -9 expression in smokers with chronic rhinosinusitis and asthma
Source: Sci Rep. 2019 Oct 25;9:15357. doi: 10.1038/s41598-019-51813-6 (PMC6814857; doi:10.1038/s41598-019-51813-6)
Supplement: Supplementary file 1 — supplementary data [file 41598_2019_51813_MOESM1_ESM.pdf]

Increased nasal matrix metalloproteinase-1 and -9 expression in smokers with chronic rhinosinusitis and asthma

Chien-Chia Huang[1,2]; Chun-Hua Wang[3]; Pei-Wen Wu[1,4]; Jung-Ru He[3]; Chi-Che Huang[1,2]; Po-Hung Chang[1,2]; Chia-Hsiang Fu[1,2] and Ta-Jen Lee[1,5]

----

1 Division of Rhinology, Department of Otolaryngology, Chang Gung Memorial Hospital and Chang Gung University, Taoyuan, Taiwan

2 Graduate Institute of Clinical Medical Sciences, College of Medicine, Chang Gung University, Taiwan

3 Department of Thoracic Medicine, Chang Gung Memorial Hospital and Medicine of College, Chang Gung University, Taoyuan, Taiwan

4 Department of Otolaryngology–Head and Neck Surgery, Chang Gung Memorial Hospital and Chang Gung University, Keelung, Taiwan

5 Department of Otolaryngology, Xiamen Chang Gung Hospital, Xiamen, China

.

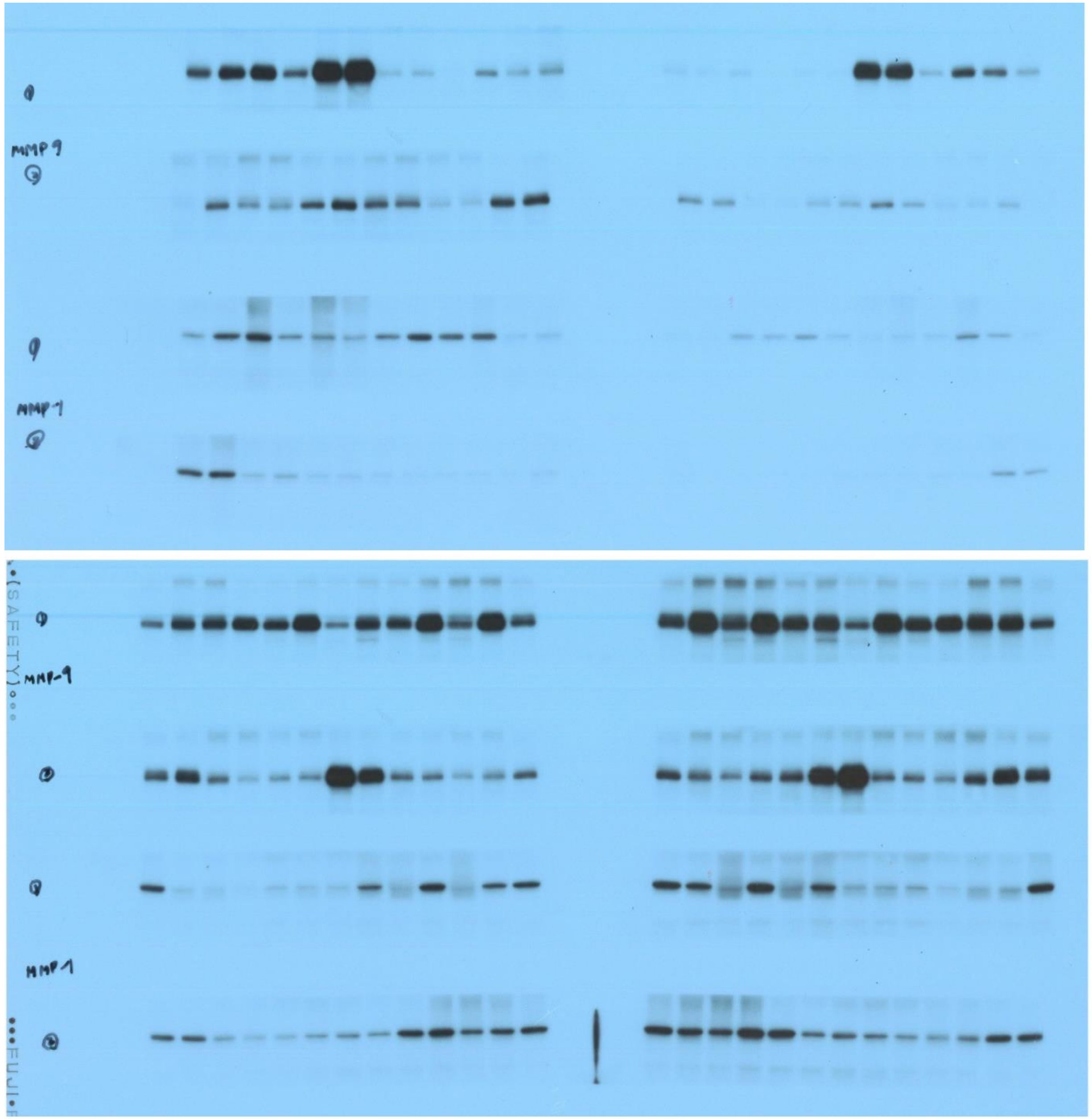

MMP-1 and -9 protein quantification by western blotting

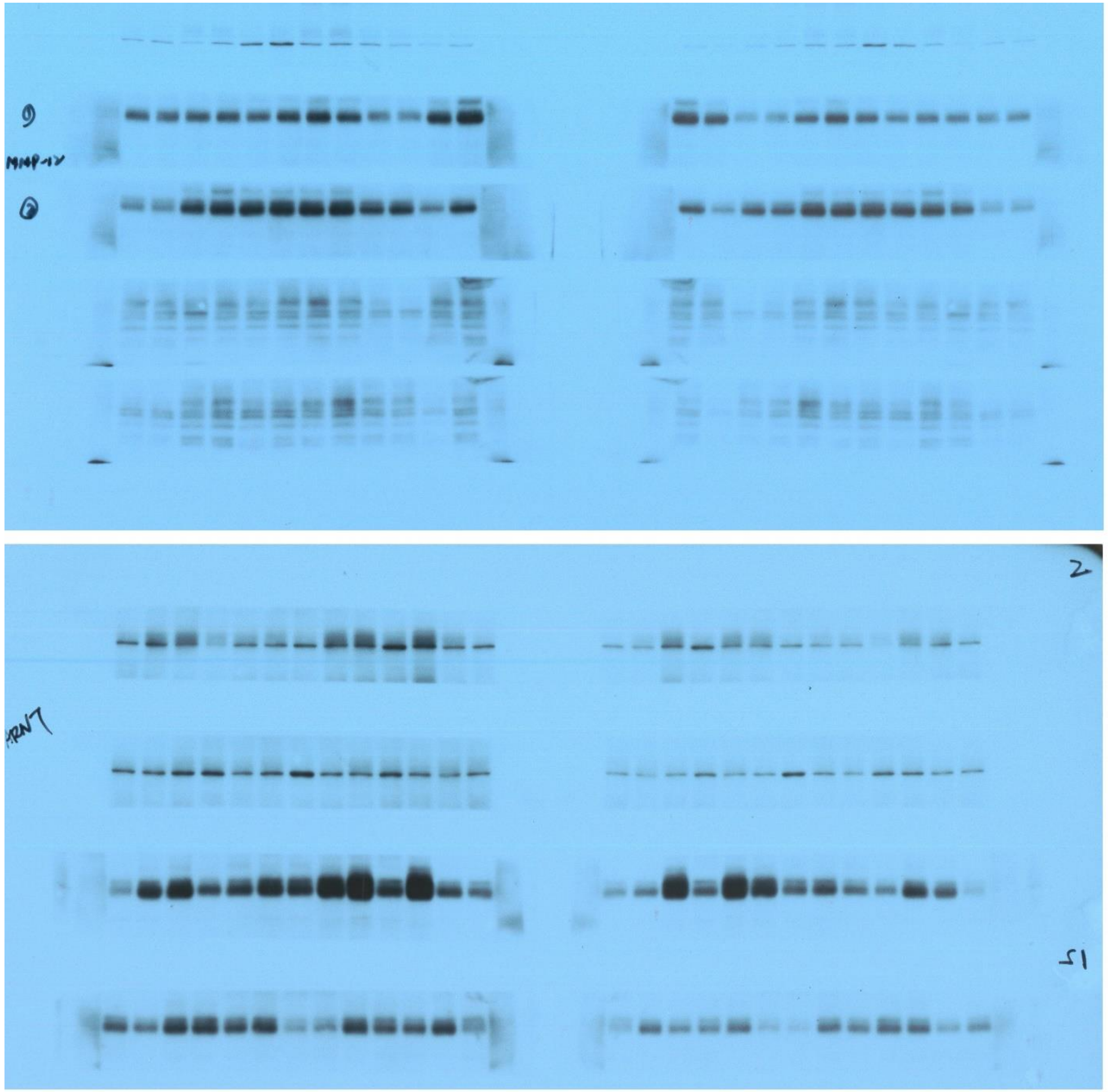

MMP-12 protein quantification by western blotting
